# Ultra-violet radiation is responsible for the differences in global epidemiology of chickenpox and the evolution of varicella-zoster virus as man migrated out of Africa

**DOI:** 10.1186/1743-422X-8-189

**Published:** 2011-04-23

**Authors:** Philip S Rice

**Affiliations:** 1 Department of Medical Microbiology, St George's Hospital, Blackshaw Road, London, SW17 0QT, UK

## Abstract

**Background:**

Of the eight human herpes viruses, varicella-zoster virus, which causes chickenpox and zoster, has a unique epidemiology. Primary infection is much less common in children in the tropics compared with temperate areas. This results in increased adult susceptibility causing outbreaks, for example in health-care workers migrating from tropical to temperate countries. The recent demonstration that there are different genotypes of varicella-zoster virus and their geographic segregation into tropical and temperate areas suggests a distinct, yet previously unconsidered climatic factor may be responsible for both the clinical and molecular epidemiological features of this virus infection.

**Presentation of the hypothesis:**

Unlike other human herpes viruses, varicella-zoster virus does not require intimate contact for infection to occur indicating that transmission may be interrupted by a geographically restricted climatic factor. The factor with the largest difference between tropical and temperate zones is ultra-violet radiation. This could reduce the infectiousness of chickenpox cases by inactivating virus in vesicles, before or after rupture. This would explain decreased transmissibility in the tropics and why the peak chickenpox incidence in temperate zones occurs during winter and spring, when ultra-violet radiation is at its lowest. The evolution of geographically restricted genotypes is also explained by ultra-violet radiation driving natural selection of different virus genotypes with varying degrees of resistance to inactivation, tropical genotypes being the most resistant. Consequently, temperate viruses should be more sensitive to its effects. This is supported by the observation that temperate genotypes are found in the tropics only in specific circumstances, namely where ultra-violet radiation has either been excluded or significantly reduced in intensity.

**Testing the Hypothesis:**

The hypothesis is testable by exposing different virus genotypes to ultra-violet radiation and quantifying virus survival by plaque forming units or quantitative mRNA RT-PCR.

**Implications of the hypothesis:**

The ancestral varicella-zoster virus, most probably a tropical genotype, co-migrated with man as he left Africa approximately 200,000 years ago. For this virus to have lost the selective advantage of resistance to ultra-violet radiation, the hypothesis would predict that the temperate, ultra-violet sensitive virus should have acquired another selective advantage as an evolutionary trade-off. One obvious advantage could be an increased reactivation rate as zoster to set up more rounds of chickenpox transmission. If this were so, the mechanism responsible for resistance to ultra-violet radiation might also be involved in reactivation and latency. This could then provide the first insight into a genetic correlate of the survival strategy of this virus.

## Background

Chickenpox epidemiology is unique among human herpes viruses. In the tropics primary infection is often delayed into later childhood whereas in temperate zones most infection occurs before leaving school. Indeed, in some tropical countries 30-50% of adults are susceptible, compared with only 5-10% from temperate areas [1].

Conventionally, transmission has been considered to occur by shedding of virus from the upper respiratory tract 1-2 days before the rash [2,3]. The papers which claim to show such virus transmission however, also conclude that the titres of virus in vesicular fluid are considerably greater than those present in the pharynx and that vesicular virus makes the greatest contribution to spread [4-6]. Indeed, the few papers cited as providing epidemiological evidence for airborne spread are either mis-quoted [7], based on case reports [8,9] or do not reflect the normal transmission environment[10]. In this regard chickenpox appears similar to smallpox, which also had a distinct winter-spring seasonal peak in incidence and was spread partly by the vesicular eruption [11].

Why such a common, global infection should be less common in children from the tropics when infections are generally more common remains unknown. Although previously suggested factors such as heat, humidity, viral interference, population density or infection with cross-protecting viruses, have been suggested as possible causes of the epidemiological differences, a unified, coherent explanation has eluded discovery [1,12]. The climatic factor which I propose to show is responsible for the geographical differences in transmission is ultra-violet radiation (UVR). Furthermore, as varicella-zoster virus (VZV) exists only in man, I propose that UVR has been involved in the co-evolution of virus as man migrated out of Africa. The evolution of varying degrees of resistance to UVR among the different genotypes [13] may also have implications for virus reactivation as zoster.

### Presentation of the hypothesis

**Figure 1:**
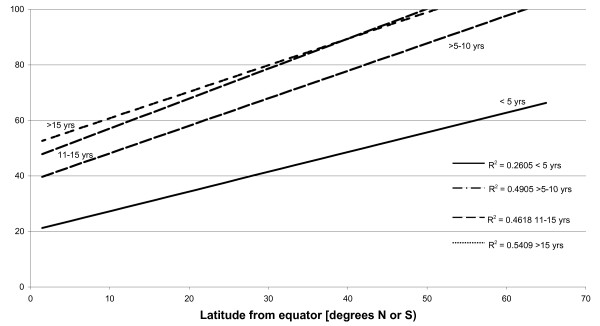
**Latitude and prevalence of VZV IgG by age**.

**Figure 2:**
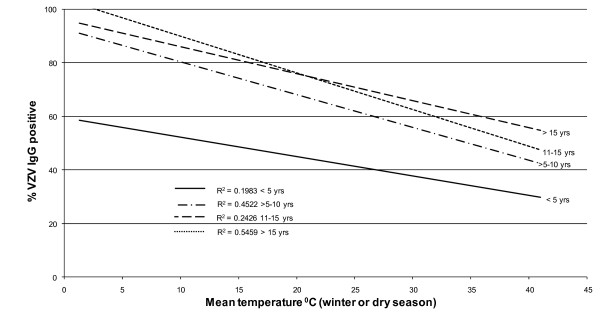
**Mean winter/dry season temperature and prevalence of VZV IgG by age**.

**Figure 3:**
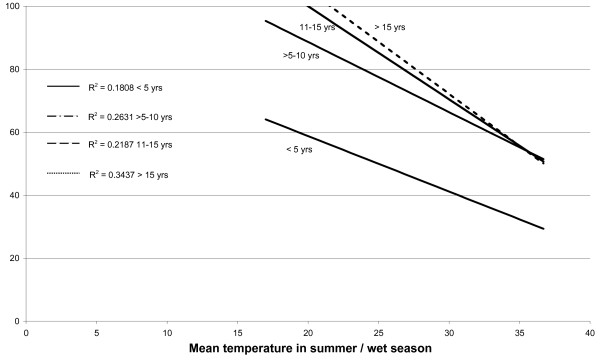
**Mean summer/wet season temperature and VZV IgG prevalence by age**.

**Figure 4:**
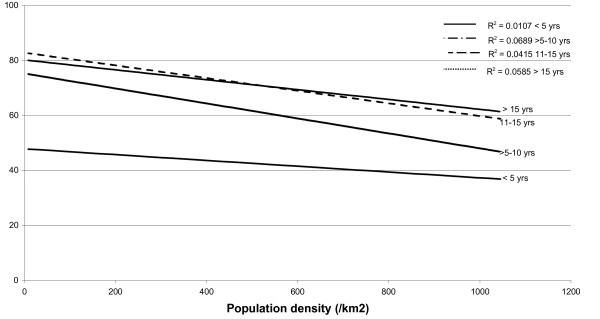
**Population density (/km2) and VZV IgG prevalence by age**.

**Figure 5:**
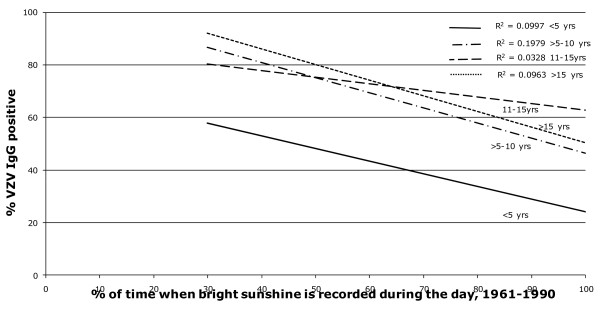
**Mean annual sunshine fraction (1961-1990), % of time with bright sunshine and age stratified VZV IgG sero-prevalence**.

A search for sero-epidemiological studies of varicella-zoster virus (VZV) using the terms "varicella", "chickenpox" and "seroepidemiology" produced a total of 25 papers. From these publications other relevant references were also located giving a total of 42 articles, reviewed in [14]. Whilst the studies were of different formats, linear regression curves of age-stratified antibody prevalence plotted against latitude showed a reasonably good fit (r^2 ^≈ 0.5) was demonstrated across all age groups of children >5 years (Figure 1). The same antibody prevalence data when plotted against temperature, rainfall, population density and sunshine, using data drawn from the World Meteorological Organisation (http://www.wmo.int) and the United Nations (http://www.fao.org/WAICENT/FAOINFO/SUSTDEV/EIdirect/CLIMATE/EIsp0002.htm), showed no consistent correlation (Figures 2, 3, 4 and 5).

Chickenpox is seasonal in temperate zones, with the highest incidence seen in winter and spring [1]. One explanation for this seasonality could be the significantly higher levels in ultra-violet radiation (UVR) of approximately 10-25-fold seen in summer in temperate zones [15], which could inactivate virus either in vesicular lesions or after their rupture. Chickenpox is not seasonal in the same way in the tropics possibly because UVR differs only by a factor of two during the year [15]. The tropics however, do experience peaks in chickenpox incidence when the climate is hot, dry and sunny with a rapid decline to very low levels during the rainy season [16-18]. This appears difficult to reconcile with UVR inactivating virus until the effects of atmospheric pollution on ambient UVR are considered. For example, the Indo-Asian haze, a continent-wide increase in air pollution during the dry season from December to April, has been shown to reduce significantly the level of ambient UVR [19]. As the Monsoon arrives, atmospheric particles and pollutants are washed out, increasing the UVR which inactivates virus more effectively. This correlates very well with the observed chickenpox incidence in Sri Lanka and south India [16,17]. Furthermore, outbreaks of varicella have been terminated in certain African countries by the arrival of the rainy season [18]. Increased atmospheric pollution might partly explain, in association with locally increased population density, why chickenpox is commoner in urban environments compared with rural communities in adjacent geographic areas [20,21].

Further support for the hypothesis derives from sequence analysis which has classified VZV into distinct genotypes. In the largest published study, 348 genotypes of VZV were given geographic locations based on where the virus was originally detected [13]. In the temperate zones which were studied (N America, Argentina, Europe, S Africa, N China, N Asia) a total of 35/259 (13.5%) genotypes were tropical. In contrast, of the 89 isolates from tropical countries/regions (India, Nepal, Bangladesh, Chad, DRC, Southern China, Western Australia, Brazil, Cote D' Ivoire, Ethiopia, Thailand, Vietnam, Zimbabwe), only 5 (5.6%) were temperate. This difference was statistically significant by Chi-square testing (p < 0.0001). Nevertheless, temperate virus genotypes, which should be more sensitive to UVR than tropical strains, and so would be out-competed in terms of transmission, have been detected in tropical areas, namely Australia, Brazil, Congo, and Mexico City. However, survival of temperate genotypes in these regions is still consistent with the hypothesis when it is considered how reducing ambient UVR allows temperate genotypes to transmit.

In Australia widespread preventative measures are taken limit exposure to UVR in schools by having large, shaded playground areas.

In urban Brazil, man-made biomass burning and in rural areas, the forest canopy and high humidity act together to reduce UVR [22].

In the Congo, the first ever demonstration of transmission of temperate virus, occurred in only one family all living in the same house, the implication being that temperate virus is rapidly inactivated by UVR after leaving the confines of the family home [23].

Finally, the detection of temperate virus genotypes from cases of chickenpox in Mexico City may be explained because it is one of the most heavily polluted cities in the world which reduces UVR, allowing temperate genotypes to survive [24].

### Proving the hypothesis

The hypothesis is biologically plausible because UVR is virucidal against many viruses, yet the effect of UVR on survival of VZV *in vitro *has never been tested [25]. However, the effect of UVR on virus transmission *in vivo *was demonstrated over 60 years ago when artificial UVR was used successfully to reduce virus transmission in US schools to limit spread of chickenpox [26]. Epidemiological evidence to support the hypothesis could be provided by correlating the transmission of different virus genotypes with ambient UV radiation. Genotyping VZV in cases of chickenpox could determine if there are seasonal differences in genotype transmission in temperate areas. The hypothesis would predict that tropical virus genotypes should predominate during summer in temperate countries since they would have the selective advantage of increased resistance to UVR.

If different genotypes of VZV possess different tolerances to UVR this could be demonstrated *in vitro *by exposing virus to UVR and quantifying the surviving virus by either plaque forming units or quantitative mRNA RT-PCR. Finally, it may also be possible to make hybrid viruses by exchanging those regions of the VZV genome which are significantly different between genotypes and determine for the first time the molecular markers that underlie transmission or reactivation of VZV.

### Implications of the hypothesis

The principal difficulty with the hypothesis is explaining how an ancestral tropical virus genotype, inherently more resistant to UVR, migrated with man out of Africa 200,000 years ago only to lose the selective advantage of resistance to UVR, form a temperate virus genotype lineage and as result become less transmissible. The solution to this paradox could be that loss of the selective advantage of resistance to UVR and reduced transmissibility was offset by an increased propensity to reactivate as zoster. This could indicate that the areas of the VZV genome which confer resistance to UVR are the same as are involved in latency and reactivation.

I suspect this to be the case because as the transmission environment is so harsh in the tropics, random mutation and natural selection should have brought about a tropical virus genotype which reactivates much more frequently to counter-act the lower transmissibility of chickenpox. The fact that the data on zoster epidemiology from tropical countries (in the pre-AIDS era) are virtually absent suggests that the tropical genotype reactivates only in severely immune suppressed individuals. Potentially it may have implications for VZV vaccine since if it was made from a tropical genotype which reactivated much less frequently, it might be possible, in years to come, to significantly reduce the disease burden from zoster.

## List of abbreviations

UVR: Ultra-violet radiation; VZV: Varicella-zoster virus

## Competing interests

The authors declare that they have no competing interests.

## Authors' contributions

Solely responsible for developing, researching and writing the hypothesis.
